# Practice variability of epinephrine administration in pediatric out-of-hospital cardiac arrest in the United States

**DOI:** 10.1016/j.resplu.2026.101454

**Published:** 2026-08-18

**Authors:** Chelsea B. Kadish, Christopher B. Gage, Jonathan R. Powell, Alexander J. Ulintz, Henry E. Wang, Christopher E. Gaw, Gregory Muller, Ashish R. Panchal

**Affiliations:** a Division of Emergency Medicine, Nationwide Children’s Hospital, Columbus, OH, United States; b National Registry of Emergency Medical Technicians, Columbus, OH, United States; c Department of Pediatrics, The Ohio State University College of Medicine, Columbus, OH, United States; d Department of Emergency Medicine, The Ohio State University College of Medicine, Columbus, OH, United States; e Department of Emergency Medicine, Section of Prehospital and Disaster Medicine, Stanford University, Stanford, CA, United States; f ImageTrend, Eagan, MN, United States

**Keywords:** Emergency medical services, Prehospital, Pediatrics, Out-of-hospital cardiac arrest, Epinephrine

## Abstract

**Background:**

Pediatric out-of-hospital cardiac arrests (pOHCA) are low frequency, high acuity events for Emergency Medical Services (EMS). The survival rate is low, with only 10% of children surviving, and few survivors having favorable neurologic outcomes. Optimal prehospital management of pOHCA includes rapid epinephrine administration, yet factors contributing to its administration are not well understood.

**Objective:**

Our study objective was to evaluate the frequency of epinephrine administration in pOHCA. Our secondary objective was to characterize the demographic and clinical characteristics associated with epinephrine administration in pediatric OHCA in the United States.

**Methods:**

In this retrospective, observational study, we analyzed data from the National Emergency Medical Services Information System (NEMSIS) dataset for the year 2021. We identified pediatric patients ages ≥1 day-old to <18 years-old from “9-1-1” activations that were treated by advanced life support (ALS) clinicians. OHCA events were identified by performance of cardiopulmonary resuscitation (CPR) or defibrillation. We used descriptive statistics to evaluate the frequency of epinephrine administration and assess demographic and clinical characteristics in this population. Multivariable logistic regression model was utilized to find characteristics associated with epinephrine administration.

**Results:**

In 2021, 8495 pOHCA events (median [IQR] age: 3.0 [0.3–13.0] years; 58.5% males) were managed by an ALS unit in the United States. Two out of every five (40.2%) pOHCA events did not receive epinephrine. Epinephrine use was more common in younger age groups (1 day-1 year OR, 1.25 [95% CI, 1.07–1.46]; 1–5 years OR, 1.19 [95% CI, 1.00–1.38]; 6–12 years OR, 1.59 [95% CI, 1.24–1.79]; referent: 13–17 years), urban population settings (OR, 2.06 [95% CI: 1.79–2.38]), incidents with scene time >10 min (OR, 1.41, [95% CI, 1.25–1.60]), advanced airway **attempt**(OR 8.33, [95% CI, 7.33–9.47]), and in those receiving defibrillation (OR 1.95, [95% CI, 1.61–2.37]). Odds of epinephrine administration were lower in public settings (OR 0.55, [95% CI, 0.47–0.65]) and in females (OR 0.83, [95% CI, 0.73–0.93]).

**Conclusion:**

Epinephrine is not given in 2 of every 5 pOHCA events. Longer scene time and additional resuscitation interventions were associated with epinephrine administration. The low rate of administration has implications for further research and reforms in the prehospital care of critically ill children.

## Introduction

Pediatric out-of-hospital cardiac arrest (pOHCA) are low frequency, high acuity events with only 10% of children surviving, and 1–3% surviving with favorable neurologic outcomes.^1^ Emergency Medical Services (EMS) clinicians are often the first to deliver care and rapid intervention is imperative to improve outcomes in this population.^2^ However, resuscitation is a set of intertwined actions and procedures and many of the interventions recommended by the American Heart Association (AHA) are difficult to achieve for EMS crews in pOHCA. Weight-based dosing and variations in equipment sizes can increase the cognitive load on an already stressful run.^3^, ^4^ Frequency of pediatric skills training may also contribute to the delivery of quality care in this population.^3^, ^5^

Yet, epinephrine administration is a key element of Pediatric Advanced Life Support (PALS), and preliminary studies suggest that children may benefit from early epinephrine administration with improvement in ROSC, 24-h survival, and survival with favorable neurologic outcomes.^6^, ^7^, ^8^, ^9^, ^10^ However, the rate of epinephrine administration and the factors contributing to delivery of epinephrine during pOHCA are not well understood. A better understanding of epinephrine administration in pOHCA is a critical step in optimizing EMS delivery of care.

Therefore, our study objectives were to (1) evaluate the frequency of epinephrine administration in pOHCA and (2) identify the demographic and clinical characteristics associated with epinephrine administration in pediatric OHCA in the United States.

## Methods

### Study design, setting, and population

We conducted a retrospective, observational study using the National Emergency Medical Services Information System (NEMSIS) public release research dataset. The American Institutes of Research Institutional Review Board deemed this study exempt from review due to the use of deidentified, aggregate data. This manuscript adheres to the best practices outlined in the Strengthening the Reporting of Observational Studies in Epidemiology (STROBE) reporting guidelines.

NEMSIS is a voluntary dataset administered by the National Highway Traffic Safety Administration (NHTSA), and serves as a national repository for standardized, aggregate EMS patient care records across the United States (US). The database contains comprehensive information on the clinical course of EMS care, including patient demographics, dispatch and response characteristics, clinical presentation, interventions, and medications. NEMSIS data reflects EMS activity from approximately 96 percent of all EMS agencies nationwide that respond to 911 calls, with the NEMSIS Technical Assistance Center receiving 75 percent of all electronic patient care reports within three days and more than 99 percent within ten days of patient contact.^11^ In 2021, NEMSIS documented more than 48 million EMS activations from over 13,000 agencies across 53 states and territories.^12^

**Fig. 1:**
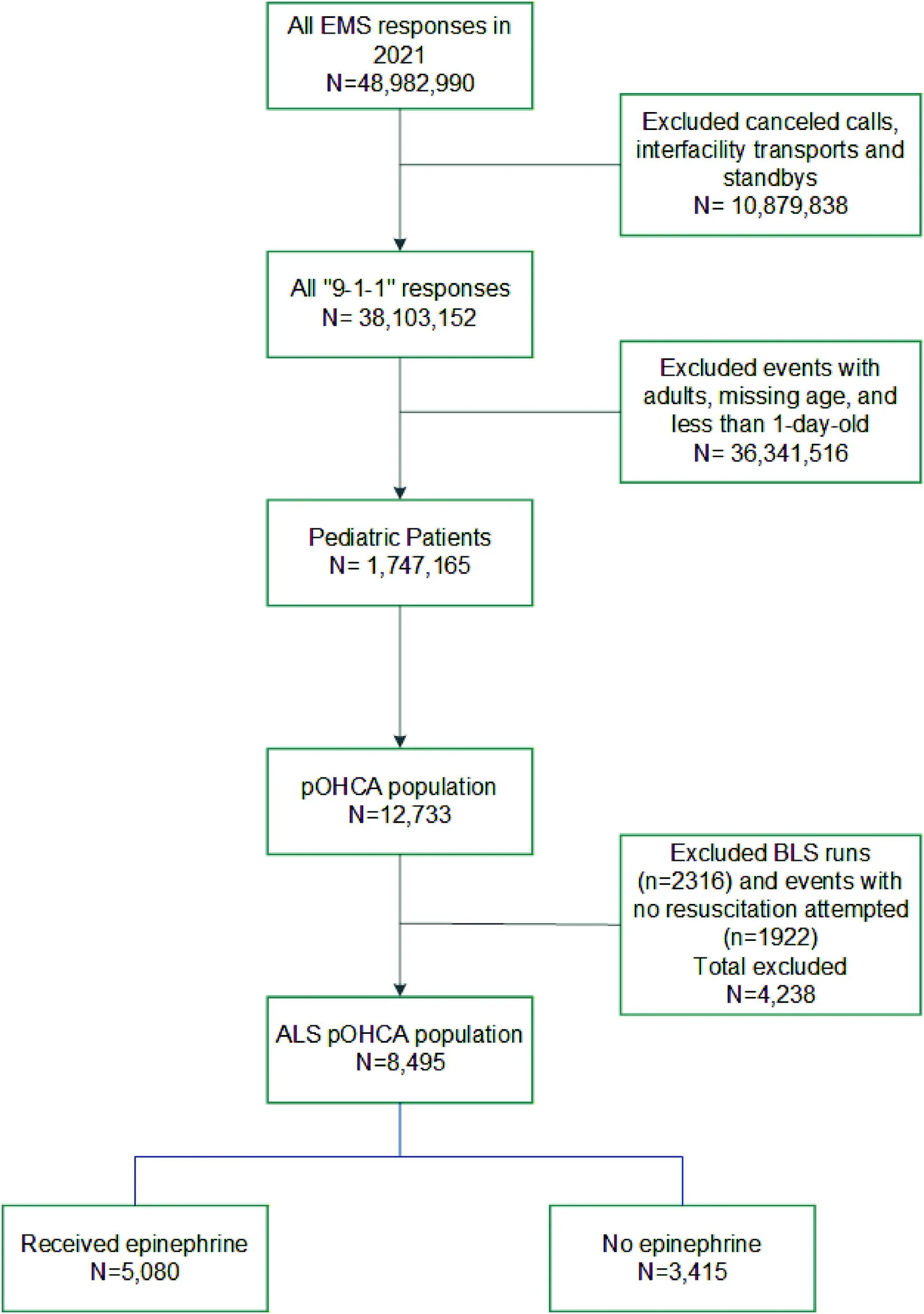
**Pediatric out-of-hospital cardiac arrests (pOHCA) receiving epinephrine**. *Abbreviations:* BLS, basic life support; EMS, emergency medical services.

We identified cases in this study using the 2021 NEMSIS public release research dataset. Among the 48 million EMS responses in 2021 (Fig. 1), we identified all 9-1-1 activations involving pediatric patients >1 day-old and <18 years-old treated by ALS providers. Canceled calls, interfacility transports, and standby events were excluded from our study cohort. Among 1,747,165 pediatric patients, we subsequently identified pOHCAs, as defined by the presence of any of the following four indicators: (1) received CPR (NEMSIS variable: e.Procedure.03), (2) presence of cardiac arrest (eArrest.03), (3) CPR attempted before or after EMS arrival (eArrest.01), or (4) performance of defibrillation (e.Procedure.03). While this search methodology is not validated, we identified our population based on methods from prior studies that have used the NEMSIS dataset to evaluate cardiac arrest.^13^, ^14^ With the primary objective to evaluate the delivery of epinephrine, we excluded pOHCAs managed by basic life support (BLS) teams, as these teams could not administer epinephrine. We further excluded patients who were not “treated.” In this study, patients were considered treated if they met any of the following four NEMSIS disposition (eDisposition.12) categories: (1) Patient Dead at Scene-Resuscitation Attempted (With Transport), (2) Dead at Scene-Resuscitation Attempted (Without Transport), (3) Patient Treated, Transferred Care to Another EMS Unit, (4) Patient Treated, Transported by this EMS Unit.

### Measures and outcomes

Our primary outcome was the frequency of epinephrine administration in pOHCA. Our secondary outcome was identifying the demographic and clinical characteristics associated with epinephrine administration in pediatric OHCA.

To characterize the demographic and clinical characteristics of pOHCAs, we report patient demographics including age (categorized into: 1 day-old to 1 year-old, 1–5 years-old, 6–12 years-old, and 13–17 years-old), sex (male and female), incident location (home/residence, healthcare facility, and public location), US Census regions (Midwest, Northeast, South, and West), NEMSIS defined urbanicity^15^ (urban and non-urban), and cause of arrest. Median EMS system response times, including response, scene, and transport times, were computed. We also characterized key clinical interventions related to pOHCA management, including advanced airways (e.g., endotracheal intubation, supraglottic airway), venous access (e.g., intravenous, intraosseous), and defibrillation.

To evaluate factors associated with epinephrine administration in pOHCA, we identified pOHCAs in our cohort that received epinephrine as described above. We subsequently identified demographic and clinical factors that were independently associated with epinephrine administration, including age, sex, incident location, urbanicity, scene time, advanced airway attempt, and defibrillation.

### Statistical analysis

Demographics, EMS response characteristics, epinephrine administration, and treatments were summarized using descriptive statistics. In comparisons of demographic and EMS characteristics among pOHCAs receiving and not receiving epinephrine, we used *t*-test and ANOVA tests for categorical variables and chi-squared tests for continuous variables. To assess outcomes associated with epinephrine administration, we performed multivariable logistic regression modeling (adjusted odds ratio [aOR], 95% confidence interval [95% CI]) to describe the associations between receiving epinephrine and population demographics, EMS scene time, and procedures performed by EMS. We utilized univariable analysis and backward selection modeling at the 0.1 alpha level to evaluate *a priori* variables for inclusion in the full model. Model fit was evaluated using the Hosmer-Lemeshow goodness of fit test (*p* > 0.05) and receiver operating characteristic curves.

To evaluate whether inclusion of trauma-related cardiac arrests influenced findings, we conducted sensitivity analyses stratified by cardiac arrest etiology and injury status. Trauma-related and non-trauma arrests were identified using eArrest.02 (Cardiac Arrest Etiology), while an additional restricted analysis included only cases where eSituation.02 (Possible Injury) was documented as “No” (not trauma). Separate multivariable logistic regression models were then constructed within each subgroup to evaluate factors associated with epinephrine administration.

## Results

In 2021, 8495 pOHCA events (median [IQR] age, 3.0 years [0.3–13.0], 58.5% males) were treated by an EMS ALS unit (Fig. 1, Table 1). Over one-third of arrests (35.5%) occurred in children aged 1 day-old to 1 year-old. Pediatric OHCA was more commonly documented by EMS ALS teams as occurring in homes (66.7%) and in urban settings (82.3%). Respiratory or asphyxia was the most common presumed cause of arrest (31.4%), followed by presumed cardiac etiology (27.9%) and trauma (16.1%). Thirty-nine percent (39.4%) of pOHCAs involved an advanced airway attempt, while a minority (11.3%) received defibrillation. The median EMS scene and transport times for pOHCA were 13.0 min (IQR 7.2–20.0) and 9.6 min (IQR, 6.0–15.3), respectively. In our cohort, two in five (40.2%, *n* = 3415) pOHCAs managed by EMS ALS units did not receive epinephrine (Table 1).

**Table 1:** Patient demographics, EMS response characteristics, and procedures stratified by epinephrine administration.

**Demographics**	**No epinephrine** ***N* = 3415 (40.2%)** ***N* (%)**	**Received epinephrine** ***N* = 5080 (59.8%)** ***N* (%)**	**Total** ***N* = 8495** ***N* (%)**
Age (median, IQR)	3.0 (0.4–14.0)	2.0 (0.3–13.0)	3.0 (0.3–13.0)
**Age categories**			
1 day–1 year	1144 (33.5%)	1875 (36.9%)	3019 (35.5%)
1–5 years	846 (24.8%)	1161 (22.9%)	2007 (23.6%)
6–12 years	465 (13.6%)	760 (15.0%)	1225 (14.4%)
13–17 years	960 (28.1%)	1284 (25.3%)	2244 (26.4%)
**Sex**			
Male	1905 (56.0%)	3047 (60.2%)	4952 (58.5%)
Female	1495 (44.0%)	2017 (39.8%)	3512 (41.5%)
*Missing*	*15*	*16*	*31*
**Census region**			
Midwest	972 (28.5%)	1207 (23.8%)	2179 (25.7%)
Northeast	227 (6.7%)	399 (7.9%)	626 (7.4%)
South	1511 (44.3%)	2332 (45.9%)	3843 (45.3%)
West	701 (20.6%)	1141 (22.5%)	1842 (21.7%)
*Missing*	*4*	*1*	*5*
**Incident location**			
Home/residence	1610 (57.5%)	2877 (73.2%)	4487 (66.7%)
Health care facility	203 (7.2%)	83 (2.1%)	286 (4.2%)
Other	988 (35.3%)	969 (24.7%)	1957 (29.1%)
*Missing*	*614*	*1151*	*1765*
**Urbanicity**			
Non-urban	820 (24.6%)	643 (13.0%)	1463 (17.7%)
Urban	2509 (75.4%)	4315 (87.0%)	6824 (82.3%)
*Missing*	*86*	*122*	*208*
**Cause of arrest**			
Cardiac presumed	683 (26.1%)	1431 (28.9%)	2114 (27.9%)
Drowning/submersion	285 (10.9%)	464 (9.4%)	749 (9.9%)
Drug overdose	97 (3.7%)	117 (2.4%)	214 (2.8%)
Electrocution	1 (0.0%)	5 (0.1%)	6 (0.1%)
Exsanguination	8 (0.3%)	16 (0.3%)	24 (0.3%)
Other	308 (11.8%)	557 (11.3%)	865 (11.4%)
Respiratory/asphyxia	775 (29.6%)	1603 (32.4%)	2378 (31.4%)
Trauma	464 (17.7%)	751 (15.2%)	1215 (16.1%)
*Missing*	*794*	*136*	*930*
**Procedures**			
Advanced airway attempt	479 (14.0%)	2867 (56.4%)	3346 (39.4%)
Vascular access	1287 (37.6%)	4398 (86.6%)	5685 (66.9%)
Defibrillation	265 (7.8%)	696 (13.7%)	961 (11.3%)
**Median time intervals, minutes (IQR)**			
EMS response time	6.0 (4.0–9.0)	6.0 (4.2–8.8)	6.0 (4.1–9.0)
EMS scene time	11.0 (6.0–17.5)	14.1 (8.2–22.0)	13.0 (7.2–20.0)
EMS transport time	10.0 (5.7–18.0)	9.3 (6.0–14.0)	9.6 (6.0–15.3)

*Abbreviation:* IQR – interquartile range.

In our multivariable analysis, epinephrine administration was significantly associated with age, sex, incident location, urbanicity, EMS scene time, advanced airway attempt, and defibrillation (Table 2). All age groups had higher odds of receiving epinephrine than adolescents (our referent population), however, the 1–5-year age group did not show statistical significance (aOR = 1.19, 95% CI [1.00, 1.38]). Epinephrine administration was more likely for incidents occurring in urban settings (aOR = 2.06, 95% CI [1.79, 2.38]) and for those with scene time over ten minutes (aOR = 1.35, 95% CI [1.25, 1.60]). Similarly, the odds of receiving epinephrine also increased in children who had other ALS interventions performed such as advanced airway attempt (aOR = 8.33, 95% CI [7.33, 9.47]) or defibrillation (aOR = 1.95, 95% CI [1.61, 2.37]). Female patients were less likely to receive epinephrine (aOR = 0.84, 95% CI [0.73, 0.93]). The odds of receiving epinephrine were also lower for events occurring public locations (aOR = 0.55, 95% CI [0.49, 0.63]) or healthcare facilities (aOR = 0.33, 95% CI [0.22, 0.40]). For children with pOHCA in healthcare facilities, it is possible that epinephrine was given prior to arrival of the EMS unit, However, this study does not include interfacility transport, meaning these are likely outpatient healthcare facilities where IV/IO access is unavailable and epinephrine likely not given prior to EMS arrival.

**Table 2:** Multivariable logistic regression evaluating factors independently associated with epinephrine administration.

**Received epinephrine**	**Adjusted odds ratio**	**95% confidence interval**	***p*-value**
**Age category**			
1 day–1 year	1.29	1.07, 1.46	0.005
1–5 years	1.19	1.00, 1.38	0.055
6–12 years	1.59	1.24, 1.79	0.000
13–17 years	Referent		
**Sex**			
Male	Referent		
Female	0.84	0.73, 0.93	0.001
**Incident location**			
Home/residence	Referent		
Public location	0.55	0.49, 0.63	0.000
Healthcare facility	0.33	0.22, 0.40	0.000
**Scene time**			
Scene time <10 min	Referent		
Scene time >10 min	1.35	1.25, 1.60	0.000
**Urbanicity**			
Non-urban	Referent		
Urban	2.06	1.79, 2.38	0.000
Advanced airway attempt	8.33	7.33, 9.47	0.000
Defibrillation	1.95	1.61, 2.37	0.000

Recognizing that epi administration could be different between trauma versus non-trauma patients, we conducted sensitivity analyses to evaluate whether removal of trauma cases effects the associations of epinephrine administration. We found consistent results across non-trauma arrests, cases where possible injury was documented as “No” (Supplemental Table 1), and primary outcome model (Table 2). Across all models, younger age groups, longer scene times, urban incidents, advanced airway attempt, and defibrillation were associated with greater odds of epinephrine administration, while arrests occurring in public locations or healthcare facilities were associated with lower odds of epinephrine administration. Female patients also demonstrated lower odds of receiving epinephrine across all sensitivity analyses.

## Discussion

Using data from the 2021 NEMSIS dataset, our study found that 40% of pediatric OHCA attended by ALS units did not receive epinephrine. Recent literature supports early administration of epinephrine in pediatric cardiac arrest showing improvement in ROSC, 24-h survival, and survival with favorable neurologic outcomes.^7^, ^8^, ^9^, ^10^ This evidence prompted a change in the 2020 PALS guidelines recommending that epinephrine is given within 5 min from the start of resuscitation in children with cardiac arrest. However, the low rate of administration of epinephrine in our study suggests that further research is needed to better understand barriers to administration for this population.

The rate of epinephrine administration was lower in our study compared to prior literature but may be a more accurate reflection of national practice. The Resuscitation Outcomes Consortium (ROC) Epidemiologic Registry reports a higher rate of epinephrine administration at 75% in their ALS treated population.^16^ However, the ROC registry consists of ten sites in the US and Canada who focus on resuscitative care and likely does not provide a complete overview of care delivered in the United States. In a pre-COVID descriptive study of pOHCA, epinephrine delivery occurred in 40% of activations, though these results are confounded by not distinguishing between those who could provide the medication (ALS teams) and those who could not (BLS teams).^17^ In our evaluation, we focused on children who receive ALS care, allowing for a more accurate estimation of true prehospital epinephrine administration rates.

Interestingly, we found that epinephrine delivery is more common in infants and school-aged children compared to adolescents, which is contrary to prior studies.^10^, ^16^, ^18^ This finding is not what we expected as adolescents are anatomically similar to adults and may be more familiar to the average EMS clinician. Additionally, vascular access is known to be more difficult in younger children, which we would have expected to decrease the likelihood of epinephrine administration.^19^, ^20^ Our findings could be explained by increased utilization of intraosseous devices, making access easier, particularly in younger children.^21^, ^22^ However, it is important to note that the high degree of collinearity between vascular access and epinephrine administration seen in our study makes it difficult to separate individual effects of each variable. It remains unclear whether the primary barrier to epinephrine administration is the challenge of obtaining IV or IO access or the clinical decision that the patient requires epinephrine. Further qualitative work could help delineate this important issue and inform targeted interventions to improve epinephrine administration. Regardless of the underlying reasons, this study confirms that a substantial proportion of pOHCAs are not receiving epinephrine, which is the desired outcome.

Another compelling finding from this study is that similar to the adult OHCA population, female sex is associated with decreased odds of receiving epinephrine.^23^, ^24^, ^25^ When evaluating the multivariable regression model, we found no interactions between sex and each individual variable in our model, including age. Researchers hypothesize that disparities in care for women may exist due to hesitation to remove clothing in order to perform interventions or place defibrillation pads. It remains unclear why female children are less likely to receive epinephrine, though the consequences are likely impactful. As with OHCA in adults, further studies examining clinician decision making regarding sex and the emotional involvement in caring for these patients are needed.

Scene time may also play a role in epinephrine administration. Our study shows that pOHCA incidents with a longer scene time have higher odds of receiving epinephrine. For adults with OHCA, current practice emphasizes remaining on scene to perform life-saving interventions as it has known benefits in survival.^26^, ^27^, ^28^ Similarly, one small study in children with OHCA shows that protocols emphasizing performance of ALS procedures on scene significantly improve time to epinephrine, ROSC, and neurologically intact survival.^29^ While rapid transport with minimal on-scene interventions is common practice in prehospital pediatric care, our data suggests that staying on scene may increase epinephrine administration which could improve outcomes in pOHCA. Additionally, our study finds that children have higher odds of receiving epinephrine when EMS clinicians perform other ALS interventions, such as advanced airway attempt, or defibrillation. It is unclear why this is the case, though other studies suggest it may be related to external factors such as training or crew size.^30^, ^31^, ^32^

Location of arrest may also influence administration of epinephrine. We found that pOHCA events occurring in public locations are less likely to receive epinephrine. Literature in adults demonstrates that arrests occurring in public locations are more likely to receive early interventions such as bystander CPR and AED use and thus are more likely to achieve ROSC and less likely to need epinephrine.^33^, ^34^ While traumatic arrests are more likely to occur in public locations,^35^, ^36^ our study did not find any difference in epinephrine administration when comparing pOHCA related to trauma or injury vs those that were non-traumatic pOHCA. However, it is important that EMS clinicians prioritize epinephrine regardless of location of the arrest. Further research is needed to understand barriers to administration of this medication in public locations.

In our study, events occurring in urban settings were more likely to receive epinephrine. This is consistent with literature in adults, which demonstrates increased utilization of epinephrine and advanced airways, increased ROSC and survival in urban locations.^37^, ^38^, ^39^ While this may be due to the fact that more rural agencies utilize BLS crews who are unable to perform these interventions, we accounted for this by only including EMS agencies that involved ALS units. Epinephrine is particularly important in rural settings where arrival times and transport times are longer. Other factors that may contribute to the disparity in epinephrine administration between urban and non-urban locations could be related to limited resources and training, but additional research is needed to assess barriers and facilitators to epinephrine administration for agencies in non-urban settings.

### Limitations

There are several limitations associated with this study. First, the estimation of cardiac arrest in this study is likely an overestimation, as NEMSIS is lacking key details to delineate this population with more accuracy. For example, there may be more than one patient care record in the dataset for patients where care was transferred to another EMS unit. In our study design, our case inclusion criteria for cardiac arrest were purposefully broad to include all possible subjects, which could result in an overestimation of pOHCA. However, this search methodology was chosen because it has been utilized in previously published papers. Second, NEMSIS does not include patient specific hospital outcomes; therefore, we do not have information on ROSC for this population, and those with early ROSC may not require epinephrine. Resuscitation time bias may also affect our findings, as interventions are more likely to occur the longer the duration of the cardiac arrest. However, we cannot account for time of arrest since no exact timing variables for arrest start, stop or duration are available. Given that our study was conducted one year after the 2020 PALS guideline recommendations, our data may not capture changes in practice with continuing guideline adoption. Variation in epinephrine administration over several years is an area for future longitudinal study.

## Conclusion

In pediatric OHCA, prompt epinephrine administration is recommended. Our study highlights that 40% of cases treated by ALS EMS units did not receive this critical medication. Specific patient characteristics, including female sex, incidents in public locations, and non-urban settings were associated with lower odds of receiving epinephrine. Odds of epinephrine administration were higher when EMS stayed on scene or provided additional interventions.

## CRediT authorship contribution statement

**Chelsea B. Kadish:** Writing – review & editing, Writing – original draft, Visualization, Project administration, Formal analysis, Data curation, Conceptualization. **Christopher B. Gage:** Writing – review & editing, Writing – original draft, Visualization. **Jonathan R. Powell:** Methodology, Formal analysis, Conceptualization. **Alexander J. Ulintz:** Writing – review & editing, Writing – original draft, Conceptualization. **Henry E. Wang:** Writing – review & editing, Writing – original draft, Visualization, Conceptualization. **Christopher E. Gaw:** Writing – review & editing, Writing – original draft, Visualization. **Gregory Muller:** Writing – review & editing, Writing – original draft. **Ashish R. Panchal:** Writing – review & editing, Writing – original draft, Visualization, Supervision, Resources, Methodology, Formal analysis, Data curation, Conceptualization.

## Declaration of competing interest

The authors declare that they have no known competing financial interests or personal relationships that could have appeared to influence the work reported in this paper.
