# Supplementary material for: Practice variability of epinephrine administration in pediatric out-of-hospital cardiac arrest in the United States
Source: Resusc Plus. 2026 Aug 18;31:101454. doi: 10.1016/j.resplu.2026.101454 (PMC13545704; doi:10.1016/j.resplu.2026.101454)
Supplement: Supplementary file 1 [file mmc1.docx]

Supplemental Table 1: Multivariable Logistic Regression Models Evaluating Factors Associated with Epinephrine Administration Among Pediatric Out-of-Hospital Cardiac Arrest Cases Stratified by Patients Without Trauma and those Without Possible Injury

| Received Epinephrine | With Trauma (includes all patients)  N=6,550 | Without Trauma  N=5,520 | Possible Injury ‘No’  N=3,853 |
| --- | --- | --- | --- |
|  | Adjusted Odds Ratio  (95% CI) | Adjusted Odds Ratio  (95% CI) | Adjusted Odds Ratio  (95% CI) |
| Age Category |  |  |  |
| 1 day - 1 year | 1.25 (1.07-1.46) ** | 1.29 (1.08-1.54) ** | 1.53 (1.24-1.89) *** |
| 1-5 years | 1.17 (1.00-1.38) | 1.17 (0.97-1.41) | 1.44 (1.14-1.81) ** |
| 6 -12 years | 1.49 (1.24-1.79) *** | 1.49 (1.20-1.85) *** | 1.62 (1.24-2.12) *** |
| 13 - 17 years | Reference | Reference | Reference |
| Sex |  |  |  |
| Male | Reference | Reference | Reference |
| Female | 0.82 (0.73-0.93) ** | 0.79 (0.69-0.89) *** | 0.77 (0.66-0.90) ** |
| Incident Location |  |  |  |
| Home/Residence | Reference | Reference | Reference |
| Public Location | 0.55 (0.49-0.63) *** | 0.51 (0.44-0.60) *** | 0.60 (0.49-0.73) *** |
| Healthcare Facility | 0.30 (0.22-0.40) *** | 0.29 (0.21-0.40) *** | 0.27 (0.19-0.38) *** |
| Scene time |  |  |  |
| Scene time < 10 minutes | Reference | Reference | Reference |
| Scene time > 10 minutes | 1.41 (1.25-1.60) *** | 1.51 (1.31-1.74) *** | 1.65 (1.40-1.94) *** |
| Urbanicity |  |  |  |
| Non-urban | Reference | Reference | Reference |
| Urban | 2.06 (1.79-2.38) *** | 2.30 (1.96-2.70) *** | 2.56 (2.10-3.12) *** |
| Advanced Airway | 8.33 (7.33-9.47) *** | 9.68 (8.34-11.23) *** | 9.65 (8.05-11.56) *** |
| Defibrillation | 1.95 (1.61-2.37) *** | 2.07 (1.68-2.56) *** | 1.93 (1.51-2.48) *** |

Supplemental Table X Footnote. Values are presented as adjusted odds ratios with 95% confidence intervals (CIs). Trauma-related and non-trauma arrest groups were defined using eArrest.02 (Cardiac Arrest Etiology). The “Possible Injury = No” group was defined using eSituation.02 (Possible Injury) coded as “No.” Models were adjusted for age category, sex, incident location, scene time, urbanicity, advanced airway attempt, and defibrillation. **p<0.01; ***p<0.001.
